# Supplementary material for: In Vitro Gut Fermentation of Whey Protein Hydrolysate: An Evaluation of Its Potential Modulation on Infant Gut Microbiome
Source: Nutrients. 2022 Mar 25;14(7):1374. doi: 10.3390/nu14071374 (PMC9003150; doi:10.3390/nu14071374)
Supplement: Supplementary file 1 [file nutrients-14-01374-s001.zip › nutrients-1591403-supplementary.pdf]

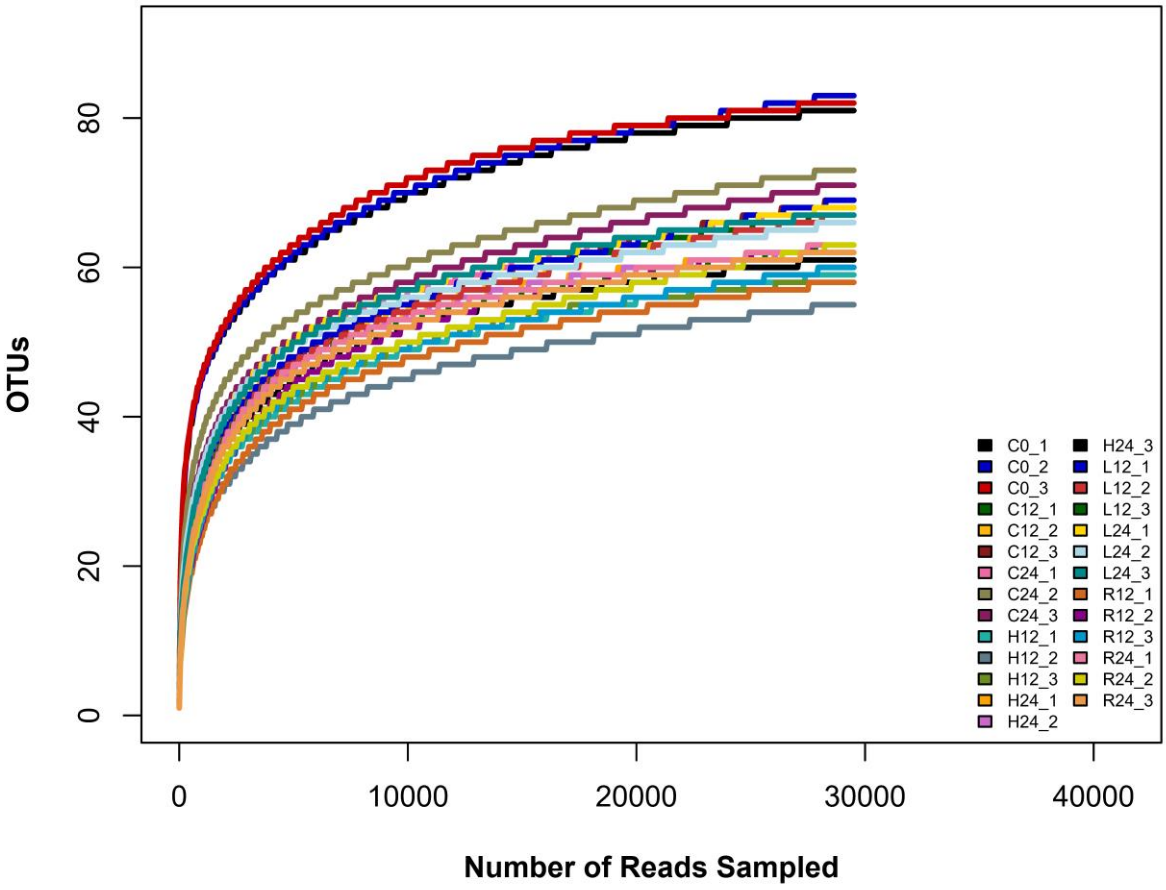

Figure S1. Rarefaction curves.

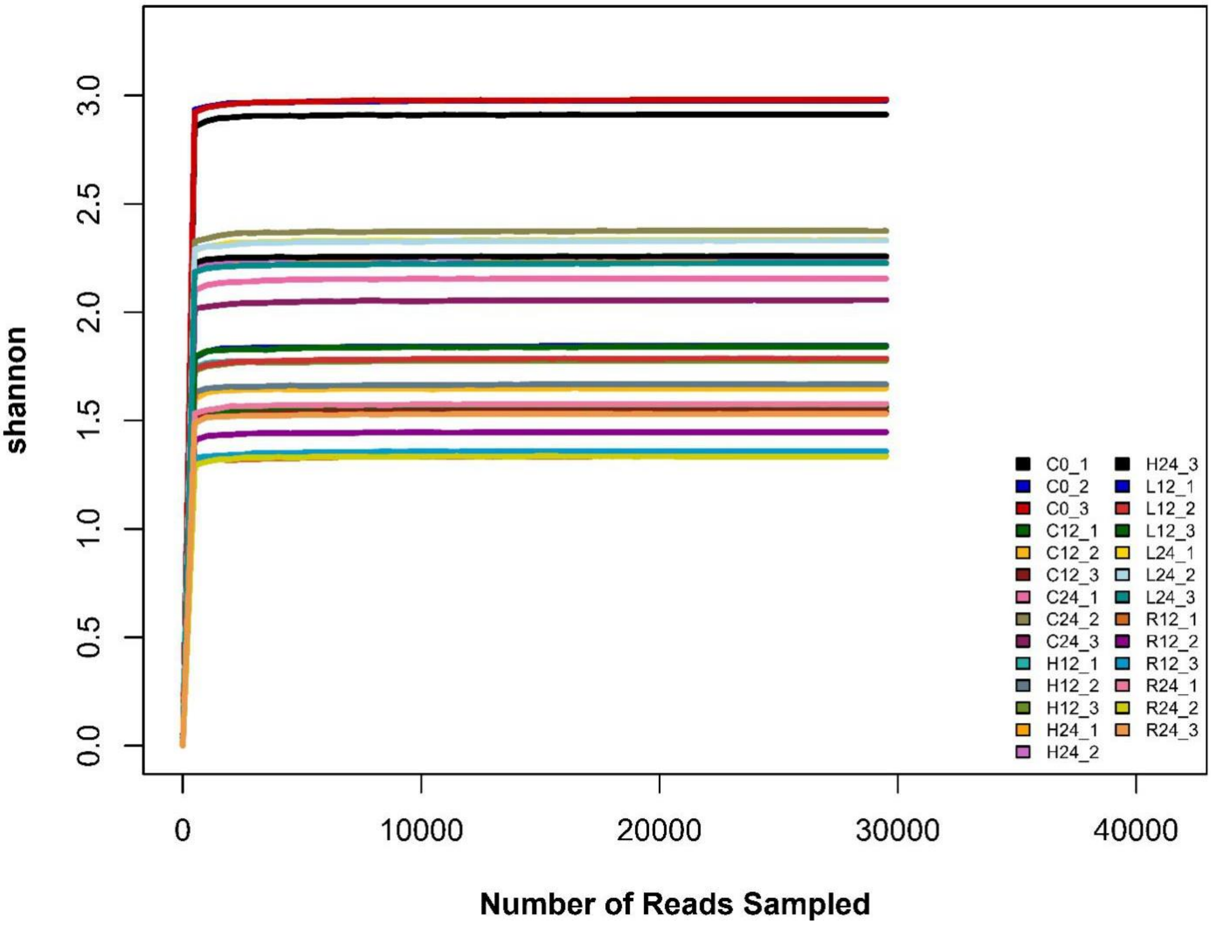

Figure S2. Shannon-Wiener curves.

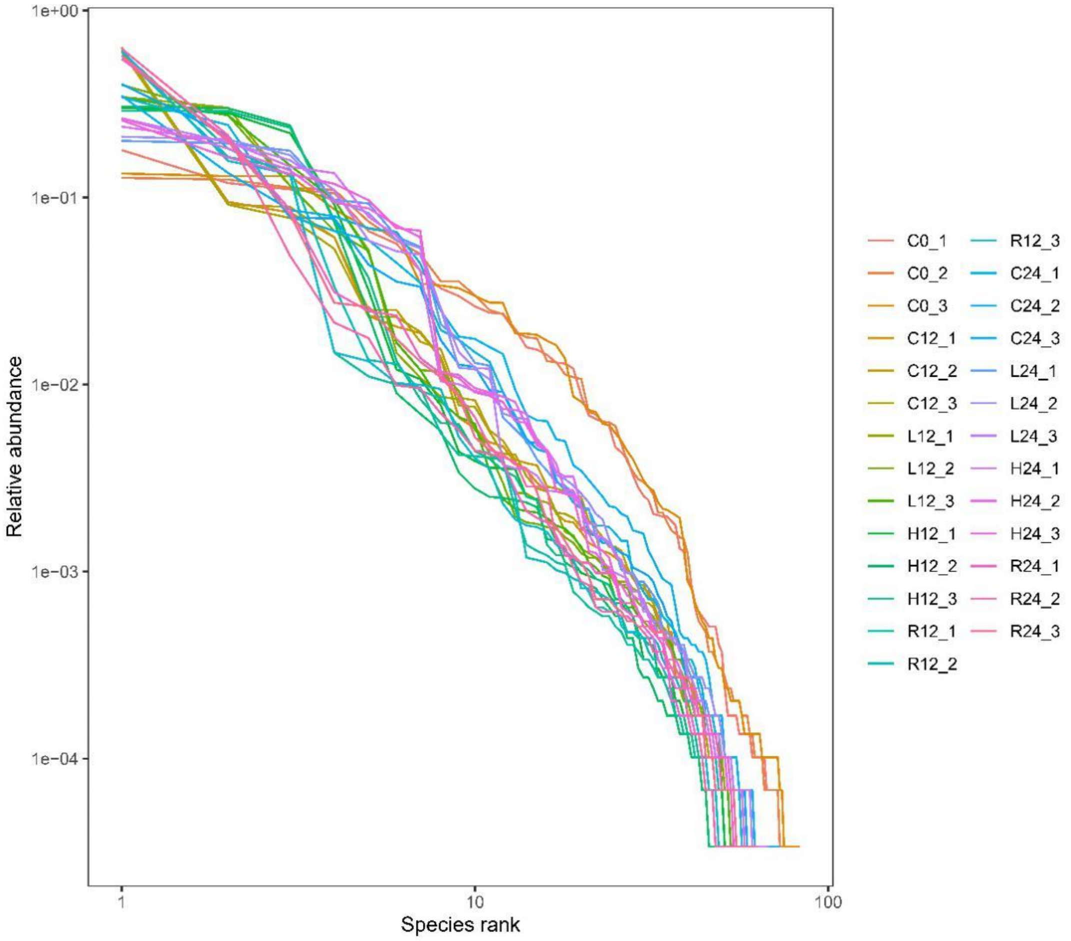

Figure S3. Rank-abundance curves

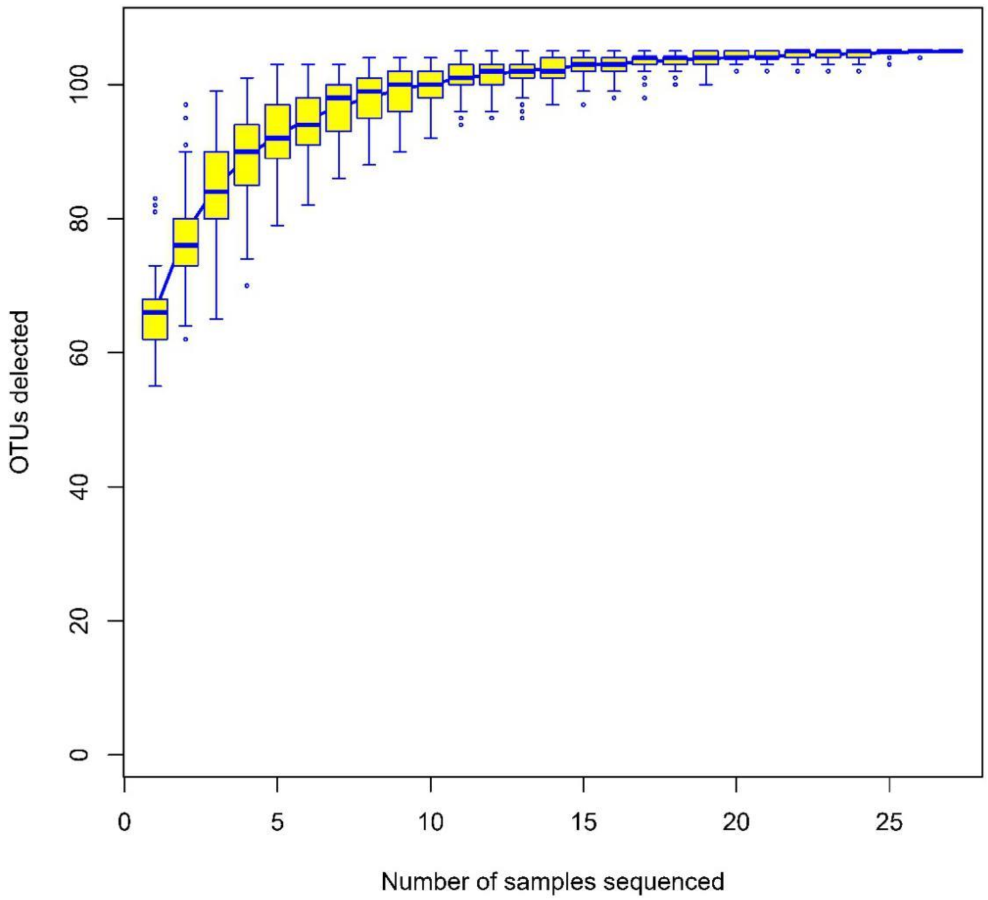

Figure S4. Species-accumulation curves

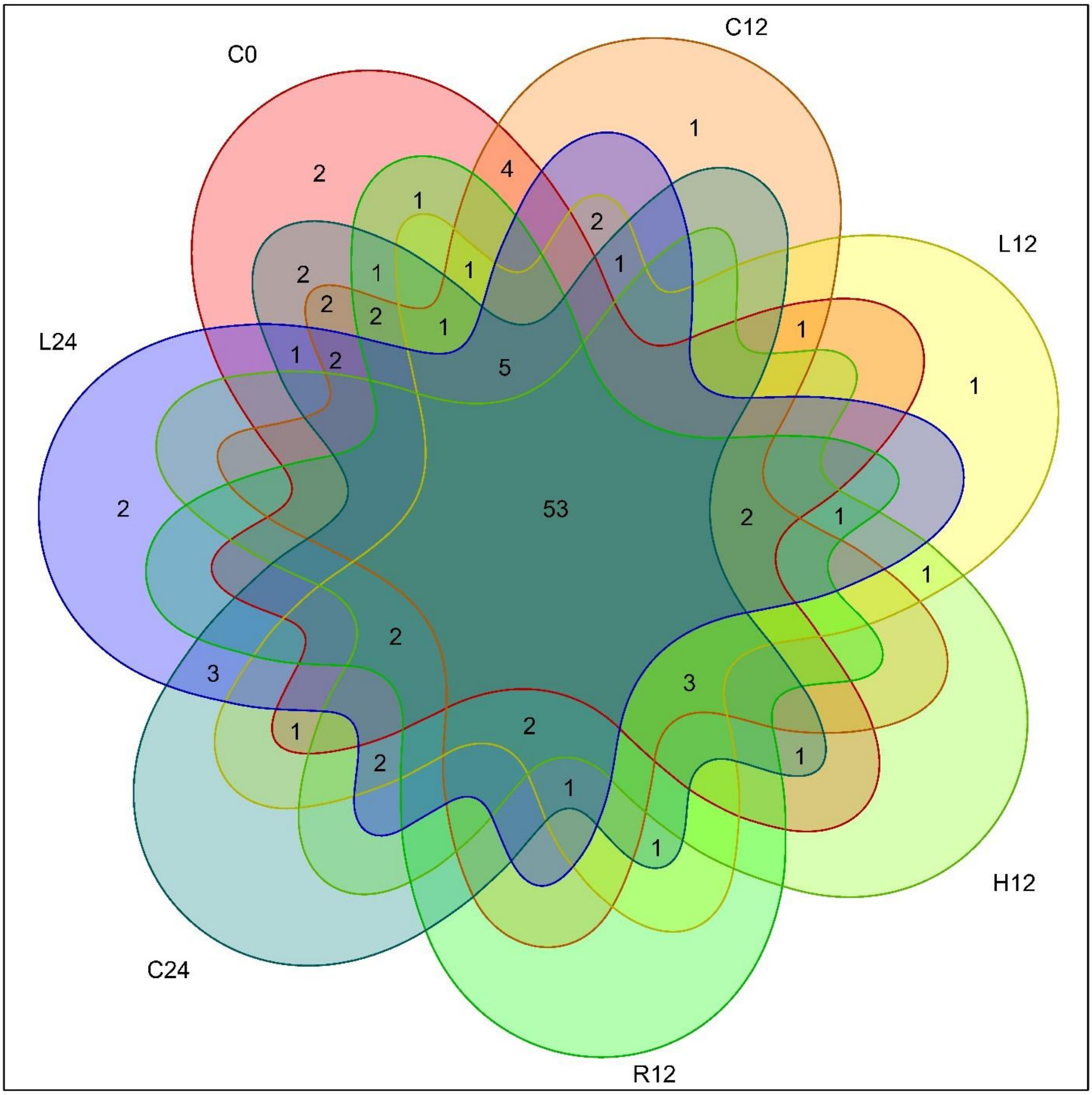

Figure S5. Venn diagram of OTUs

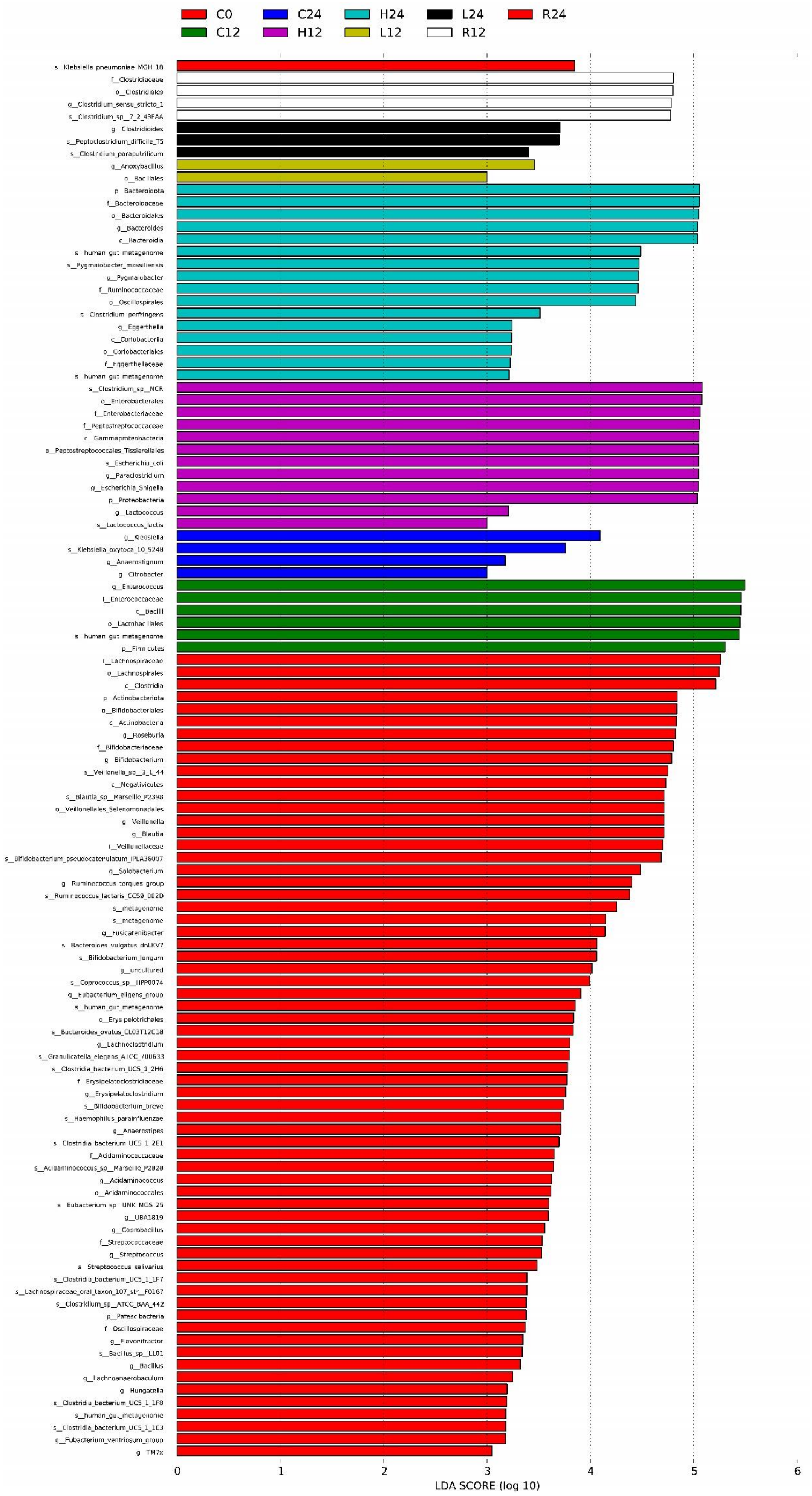

Figure S6. The histogram of LDA effect size analysis
